# Aging affects the number and morphological heterogeneity of rat phrenic motor neurons and phrenic motor axons

**DOI:** 10.14814/phy2.15587

**Published:** 2023-01-25

**Authors:** Matthew J. Fogarty, Gary C. Sieck

**Affiliations:** ^1^ Department of Physiology & Biomedical Engineering Mayo Clinic Rochester Minnesota USA

**Keywords:** aging, motor neurons, neurodegeneration, respiratory system

## Abstract

Diaphragm muscle (DIAm) motor units comprise a phrenic motor neuron (PhMN), the phrenic nerve and the muscle fibers innervated, with the size of PhMNs and axons characteristic of motor unit type. Smaller PhMNs and their axons comprise slow (type S) and fatigue‐resistant (type FR) DIAm motor units, while larger PhMNs and their axons comprise more fatigable (type FF) motor units. With aging, we have shown a loss of larger PhMNs, consistent with selective atrophy of type IIx/IIb DIAm fibers and reduced maximum DIAm force. In the present study, we hypothesized that with aging there is a loss of larger myelinated phrenic α motor axons. Female and male young (6 months) and old (24 months) Fischer 344 rats were studied. PhMNs were retrogradely labeled by intrapleural injection of 488‐conjugated CTB. The phrenic nerves were excised ~1 cm from the DIAm insertion and mounted in resin, and phrenic α motor axons were delineated based on size (i.e., >4 μm diameters). In older rats, the number of larger PhMNs and larger phrenic α motor axons were reduced. There were no differences in non‐α axons. In addition, there was evidence of demyelination of larger phrenic α motor axons in older rats. Together, these findings are consistent with the selective age‐related vulnerability of larger PhMNs and denervation of type FF motor units, which may underlie DIAm sarcopenia.

## Graphical abstract

We empirically determined that size distributions of phrenic motor neurons matched those of phrenic axons, within the same animals. In old age rats, where larger phrenic motor neurons die, larger phrenic axons were also lost. These results are consistent with functional disturbances of old age respiratory function.

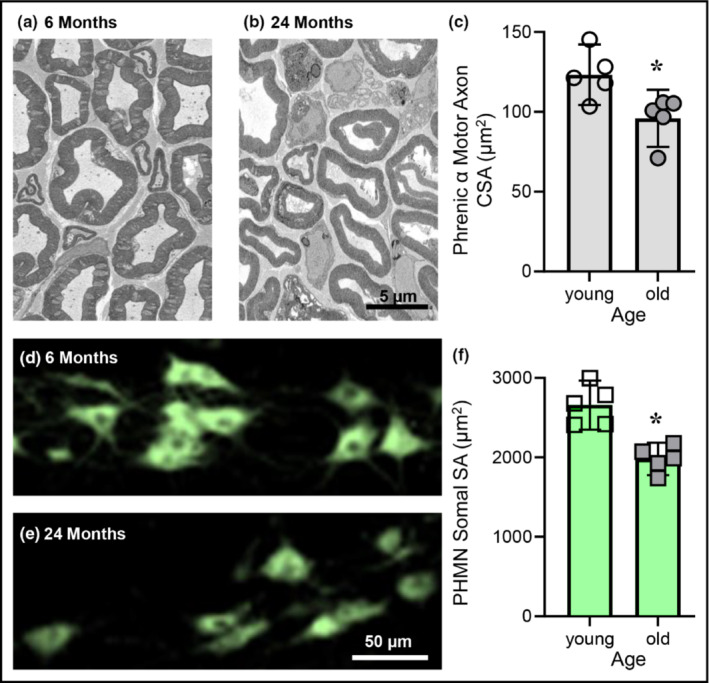

## 1 INTRODUCTION

Motor units comprise an α motor neuron and the complement of skeletal muscle fibers that it innervates. Motor units exhibit marked structural and functional differences that define different motor unit types (Cullheim & Ulfhake, 1979; Fogarty & Sieck, 2019a). Slow (type S) and fast fatigue‐resistant (type FR) motor units comprise smaller motor neurons that innervate type I (expressing the slow myosin heavy chain [MyHC_slow_] isoform) and type IIa (expressing the MyHC_2A_ isoform) muscle fibers, respectively (Burke et al., 1973; Edstrom & Kugelberg, 1968; Fournier & Sieck, 1988; Sieck et al., 1996). In the diaphragm muscle (DIAm), type I and IIa fibers generate lower maximum tetanic force and are fatigue resistant (Geiger et al., 2000). In contrast, more fatigable fast (type FF) motor units comprise larger motor neurons that innervate type IIx/IIb (expressing MyHC_2X_ and/or MyHC_2B_ isoforms) muscle fibers that generate greater maximum force yet fatigue with repetitive activation (Burke et al., 1973; Edstrom & Kugelberg, 1968; Fournier & Sieck, 1988; Geiger et al., 2000; Schiaffino & Reggiani, 2011; Sieck et al., 1996).

The size dependence of motor unit recruitment is related to the intrinsic electrophysiological properties of motor neurons – the Size Principle (Henneman et al., 1965a, 1965b). Smaller motor neurons have lower membrane capacitance and higher input resistance and are thus more excitable and the first to generate an action potential in response to excitatory input (Henneman et al., 1965b). Smaller motor neurons also have smaller axon diameters (Clamann & Henneman, 1976; Cullheim & Ulfhake, 1979; Dick et al., 1987) with slower axonal conduction velocities, a key observation in the formulation of the Size Principle (Henneman et al., 1965b). A wide variety of other morphological and physiological properties of motor neurons are size‐dependent, including dendritic arborization (Cullheim et al., 1987; Fogarty, Mu, et al., 2020; Fogarty, Mu, et al., 2021; Leroy et al., 2014; Ma & Vacca‐Galloway, 1991), innervation ratio (number of muscle fibers innervated per motor neuron) (Burke & Tsairis, 1973; Manuel et al., 2019), and oxidative capacity (Miyata & Kawai, 1991, 1992; Sickles & McLendon, 1983; Sickles & Oblak, 1984). Although the MyHC‐dependent mechanical and fatigue properties of different muscle fiber types is the major determinant of motor unit type (Geiger et al., 2000; Schiaffino & Reggiani, 2011; Sieck et al., 1996), a wide range of other molecular markers in motor neurons have been proposed for motor unit discrimination, but remain largely unverified (Fogarty, 2018).

In the DIAm, motor units comprise a phrenic motor neuron (PhMN) in the cervical spinal cord, an α phrenic motor axon, and the innervated DIAm muscle fibers (Krnjevic & Miledi, 1958). Based on measurements of phrenic axonal conduction velocity during DIAm motor unit recruitment, the Size Principle was confirmed (Dick et al., 1987) and appears tailored to meet the functional demands of breathing, which requires recruitment of only type S and FR units (Fogarty & Sieck, 2019a, 2019b; Sieck & Fournier, 1989). More forceful straining expulsive DIAm behaviors are far less frequent and require recruitment of FF motor units (Fogarty & Sieck, 2019a, 2019b; Sieck & Fournier, 1989). For motor units in the DIAm, these functional distinctions are relevant under pathophysiological conditions affecting PhMNs and/or DIAm muscle fibers, such as early‐onset spasticity (Brandenburg et al., 2020; Fogarty et al., 2022; Fogarty, Brandenburg, & Sieck, 2020), cervical spinal cord injury (Khurram et al., 2019; Miyata et al., 1995) and aging (Fogarty, Gonzalez Porras, et al., 2019; Fogarty, Mantilla, & Sieck, 2019; Fogarty, Marin Mathieu, et al., 2020; Fogarty, Omar, et al., 2018; Gosselin et al., 1994; Khurram et al., 2018).

Previously, we observed size‐dependent differences in dendritic arborization among PhMNs (Brandenburg et al., 2020; Fogarty, Omar, et al., 2018), as well as for the distribution of glutamatergic synaptic inputs (Rana et al., 2019), and the expression of glutamatergic receptors (Rana et al., 2020). Although previous studies have suggested that axon diameters are correlated with motor neuron size (Clamann & Henneman, 1976; Cullheim & Ulfhake, 1979; Dick et al., 1987), it is currently unknown if the distribution of phrenic motor axon diameters (or cross‐sectional areas – CSA) reflect the distribution of PhMN size (e.g., somal surface area). In the rat phrenic nerve, it has been reported that there are ~350–400 myelinated axons (Gottschall, 1981; Inestrosa & Alvarez, 1988; Langford & Schmidt, 1983; Smith & Rosenheimer, 1984), but analyses of the frequency distribution of phrenic axon CSA have largely focused on comparisons of the impact of dorsal and ventral root lesions and the relative composition of motor and sensory axons (Langford & Schmidt, 1983; Nair et al., 2017). Of note, it has been estimated that ~20%–40% of all myelinated phrenic axons do not derive from PhMNs and belong to either γ motor neurons (<5%) or sensory axons (Gottschall, 1981; Landau et al., 1962; Langford & Schmidt, 1983; Nair et al., 2017). In the rat, γ motor neuron and sensory axons can be discriminated by their smaller axon diameters (>4.0 μm), leaving an overall estimate of ~230–260 α PhMNs based on myelinated axons with diameters >4.0 μm (Gottschall, 1981; Landau et al., 1962; Langford & Schmidt, 1983). This number of larger myelinated axons generally matches the number of retrogradely labeled PhMNs (~250 PhMNs) (Fogarty, Omar, et al., 2018; Fogarty, Rana, et al., 2021; Mantilla et al., 2009). Previously, we found a disproportionate loss of larger PhMNs in older Fischer 344 (F344) rats (Fogarty, Omar, et al., 2018). In the present study, we hypothesize that there is a corresponding age‐related decrease in the number of larger phrenic α motor axons.

## 2 METHODS

### 2.1 Ethical approval, animals and animal anesthesia

All protocols were approved by the Mayo Clinic Institute Animal Care and Use Committee (IACUC #A57714) and complied with National Institutes of Health (NIH) and American Physiological Society guidelines. A total of 10 pathogen‐free 6‐ (young) and 24‐month (old) F344 rats (6 month: 205–340 g; 24 month: 250–460 g; five females, five males), obtained from Charles River and the National Institute of Aging colony were used in this study. Rats were housed in pairs under a 12 h:12 h light–dark cycle with ad libitum access to food and water. The animals were allowed at least 1 week to acclimate to these conditions before any experiments were performed. At the terminal experiment, animals were deeply anesthetized with intraperitoneal injection of ketamine (80 mg/kg) and xylazine (10 mg/kg) and euthanized via exsanguination.

### 2.2 PhMN labeling

Three days prior to terminal experiments, rats underwent bilateral intrapleural injections of CTB (10 μl 0.2% Alexa 488‐conjugated CTB in two injections per side), in a manner identical to previous studies (Fogarty, Rana, et al., 2021; Mantilla et al., 2009; Prakash et al., 1993). After 3 days, this intrapleural injection technique completely labels PhMNs via retrograde transport.

### 2.3 Tissue processing

Terminal procedures were performed in deeply anesthetized rats, with the thoracic portion of the phrenic nerve (~1 cm from the DIAm insertion) excised and placed in Trump's fixative (1% glutaraldehyde and 4% formaldehyde in 0.1 M PBS, pH 7.2). These segments were then rinsed in 0.1 M PBS (pH 7.2), followed by 30 min postfix in phosphate‐buffered 1% osmium tetroxide (OsO4). After rinsing in distilled water, samples were stained with 2% uranyl acetate for 15 min at 60°C, rinsed, dehydrated in progressively higher concentrations of ethanol and 100% propylene oxide, and embedded in Spurr's resin.

Following excision of the phrenic nerve, rats were perfused intracardially with a 0.1 M phosphate‐buffered saline (PBS), euthanized via exsanguination, and then perfused with 4% paraformaldehyde in 0.1 M PBS. The C_1_–C_6_ segment of the spinal cord was then dissected, post‐fixed in 4% paraformaldehyde for 24 h, and then transferred to 25% sucrose in 0.1 M phosphate buffer for 3 days at 4°C. Spinal cord tissue embedded in cryomoulds (VWR), was cut in longitudinal horizontal sections at 70 μm and mounted on Tissue Tack slides (Polysciences) that were pre‐coated with Cell‐Tak adhesive (Becton Dickinson Lab Ware). Cut sections were then mounted in prolong gold anti fade media (Cat# P36934; ThermoFisher), cover‐slipped and stored until imaging, within 3 weeks of processing.

### 2.4 Confocal imaging

Labeled PhMNs in the cervical spinal cord sections were visualized with an Olympus FV2000 laser confocal microscope (Olympus Life Sciences Solutions) equipped with an argon (488 nm) laser. Three‐dimensional confocal imaging techniques of PhMNs have been previously reported in detail (Fogarty, Omar, et al., 2018; Fogarty, Rana, et al., 2021; Prakash et al., 1993). Briefly, all images were acquired at 16‐bit resolution in an array of 1024 × 1024 pixels using a 60× Plan Apo oil‐immersion objective (NA 1.40) with a step size of 1 μm (voxel dimensions: 0.207 × 0.207 × 1.0 μm).

### 2.5 PhMN quantifications

We assessed each PhMN whose entire volume was contained within the confocal *z*‐stack. Based on multiple morphological properties of adult rodent PhMNs (Brandenburg et al., 2020; Fogarty, Omar, et al., 2018), we did not expect to observe a sex difference, so data from males and females were collapsed into single groups. Somal surface area was estimated in a stereological manner (Slomianka, 2020), using the formula for prolate spheroids, obtaining the long and short axis of every 5th PhMNs at their largest eminence using Image J (Schneider et al., 2012), in a manner identical to prior PhMN studies (Fogarty, Omar, et al., 2018; Fogarty, Rana, et al., 2021; Prakash et al., 1993; Rana et al., 2019).

### 2.6 Phrenic axon quantification and classification

Semi‐thin sections (200 nm) comprising the entire phrenic nerve was used to quantify myelinated phrenic motor axon number and CSA. All myelinated axons were counted and the CSA of each axon determined by circumscribing each axon, including the myelin sheath using Image J (Schneider et al., 2012). Previously, categorization of myelinated fibers into α or non‐α (presumably γ for heavily spindled motor pools) has been attempted in ventral nerve rootlets, spinal cord white matter and in peripheral nerves themselves. In adult cats, mean L7 ventral root α fiber diameter was ~8–9 μm, compared to mean γ diameters of ~2–3 μm (Fabricius et al., 1994). In a dual study of HRP‐labeled cat sciatic motor neurons and their constituent axons, all α motor axon diameters were >4 μm in the white matter (Cullheim, 1978). In dog phrenic nerves, 90% of myelinated afferent fibers were classified as having diameters of ~9–12 μm, with a smaller subpopulation ranging in diameter from 2 to 4 μm (Landau et al., 1962). Based on the empirical results of prior investigations (Cullheim, 1978; Fabricius et al., 1994; Fazan et al., 2009; Landau et al., 1962), and the assumptions used in other efforts categorizing myelinated fibers (Eccles & Sherrington, 1930; Gottschall, 1981; Henneman, 1957; Langford & Schmidt, 1983; Mitteregger, 1979; Stankovic et al., 2005), myelinated phrenic axons were distinguished as phrenic α motor axons based on diameters of >4 μm.

Serial blockface EM was carried out on regions of interest determined from semi‐thin survey sections, with the distance between each serial image being 0.1 μm. Phrenic α motor axons were then identified within each serial blockface image. To sample the myelination of axons, a Cavalieri volume estimation scheme was employed (Fogarty et al., 2013, 2015; Fogarty, Rana, et al., 2021; Nyengaard & Gundersen, 2006; Prakash et al., 1994; Slomianka, 2020), with sampling split into three *z*‐slices each 1 μm apart. Within these images, the CSA of the myelinated axon boundary and the axoplasmic axon CSA were measured. To assess myelination in phrenic α MN axons and non‐α axons, the myelin CSA was determined by subtracting the axoplasmic CSA from the total axon CSA, with the mean taken across the three *z*‐slices for each axon.

### 2.7 Statistical analyses

Based on previous reports (Inestrosa & Alvarez, 1988), three animals are required to detect a biologically relevant loss of >20% in phrenic axon number with age, determined by power analysis (*α* = 0.05, *β* = 0.80). Based on the variability of PhMN number and size in F344 rats (Fogarty, Omar, et al., 2018; Fogarty, Rana, et al., 2021) an *n* = 5 is necessary to detect changes of >20% in PhMNs. Thus, an *n* = 5 was chosen. Statistical analysis was performed using Prism 8 (Graphpad Software) with two‐way ANOVAs and Bonferroni post‐tests used to compare PhMNs and phrenic axons between ages and experimental technique. Kolmogorov–Smirnov tests were used to determine differences in cumulative frequency distributions in phrenic α motor axon CSA with age and to compare *Z*‐scores of PhMN SA and phrenic α motor axon CSA within young rats. All continuous data were assessed for normality with Shapiro–Wilk tests. A priori it was determined that within a particular data set, any data point outside 2.5 standard deviations from the mean was excluded from further analysis. Significance was set as *p* < 0.05, and all data are presented as means ±95% confidence intervals (CI), unless otherwise stated. Previously, we showed that there are no sex differences in PhMN properties (Brandenburg et al., 2020; Fogarty, Omar, et al., 2018; Fogarty, Rana, et al., 2021). Accordingly, data for females and males were combined for analyses.

## 3 RESULTS

### 3.1 Distributions of PhMN surface areas and phrenic α motor axon cross‐sectional areas

**FIGURE 1:**
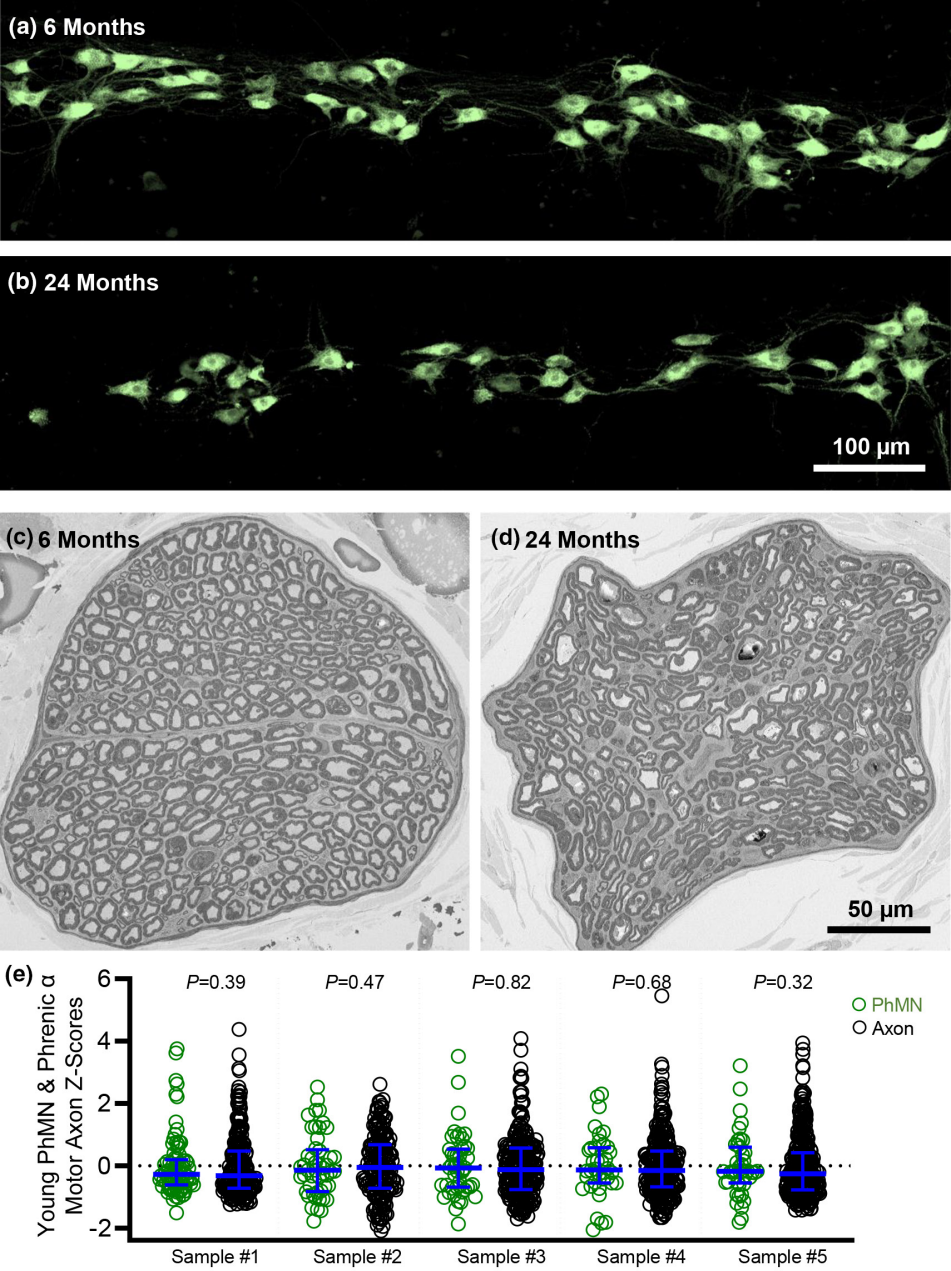
Assessment of phrenic motor neurons (PhMNs) and phrenic α motor axons in young and old rats. (a, b) Example fluorescent photomicrographs of PhMNs of young (6 months) and old (24 months) F344 rats, respectively. (c, d) Example survey EM photomicrographs of phrenic α motor axons of young (6 months) and old (24 months) F344 rats, respectively. (e) Scatterplots show *Z*‐scores (median ± inter‐quartile range) for PhMN surface area SA (green circles) and phrenic α motor axon cross‐sectional area (CSA) (black circles) from each 6‐month‐old rat sample. Kolmogorov–Smirnov comparisons with each *z*‐distribution of each young rat showing no differences in size heterogeneity between PhMNs and phrenic α motor axons. Each mean comprises high‐magnification PhMN SA estimates from a minimum of 30 (every 5th) PhMNs/rat/age and high‐magnification phrenic α motor axon CSA estimates from each counted axon.

There were no differences in the distribution of *Z*‐scores of PhMN surface areas and phrenic α motor axon cross‐sectional areas between young versus old F344 rats (*p* > 0.05, Kolmogorov–Smirnov tests for comparisons of *Z*‐scores within a rat; Figure 1). There were no differences in the minimum range of all myelinated axon diameters between young (1.34 ± 0.41 μm) and old (1.32 ± 0.54 μm) F344 rats (*p* = 0.92, Student's unpaired *t*‐test). There were no differences in the maximum range of all myelinated axon diameters between young (23.08 ± 1.25 μm) and old (22.68 ± 1.61 μm) F344 rats (*p* = 0.94, Student's unpaired *t*‐test).

### 3.2 Number of phrenic α motor axons and retrogradely labeled PhMNs

**FIGURE 2:**
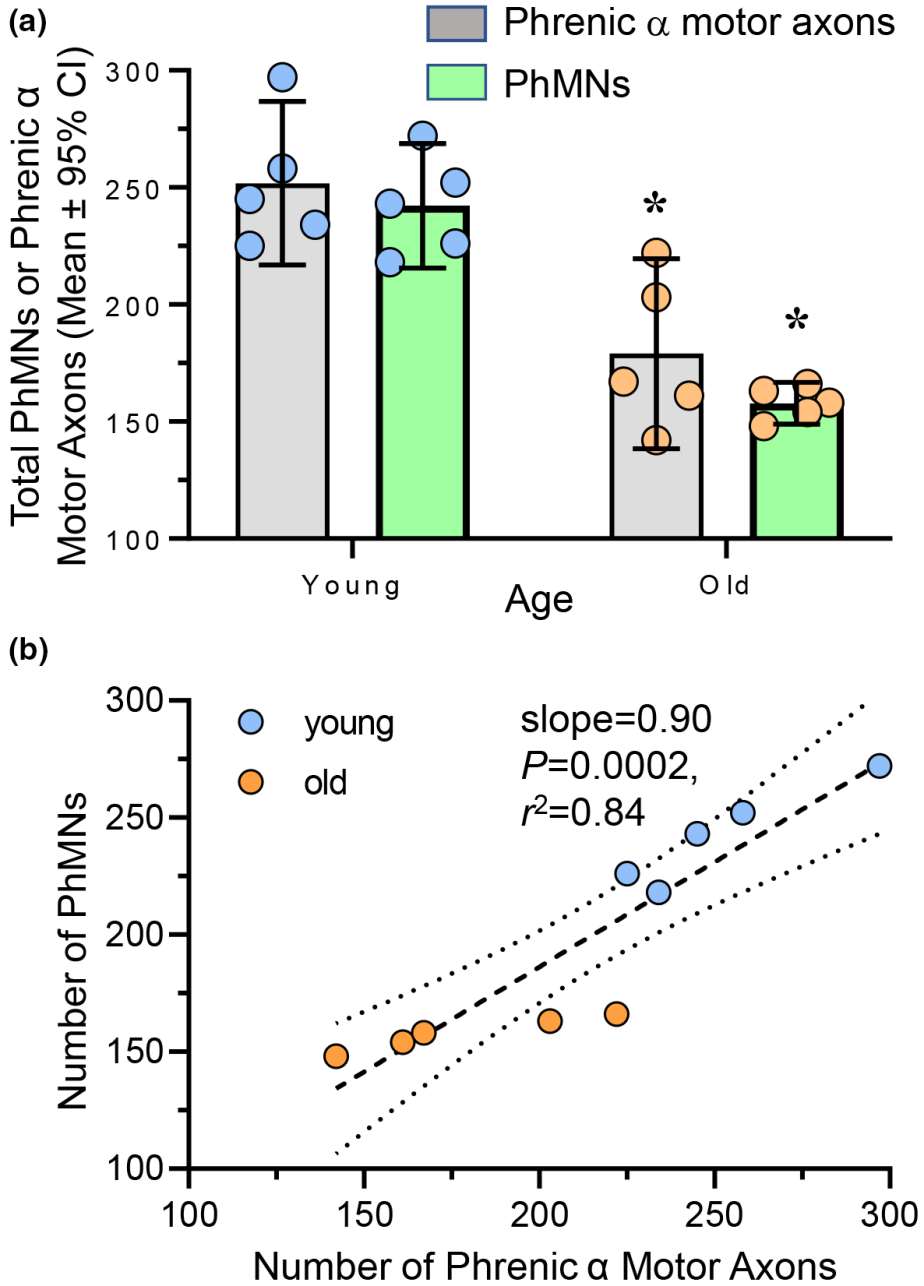
Loss of phrenic motor neurons (PhMNs) and phrenic α motor axons in old rats. (a) Scatterplot shows reduced number of PhMNs (green bars) and phrenic α motor axons (gray bars) in old (blue symbols) compared to young (orange symbols) F344 rats. Within each age group, there was good agreement between PhMN and phrenic α motor axon numbers. Two‐way ANOVA with Bonferroni post hoc test, *p* < 0.05, *n* indicated by symbol. (b) *XY* plot shows the number of PhMNs plotted against the number of phrenic α motor axons within each rat. Linear regression shows excellent agreement between the two estimates (slope = 0.90, *p* = 0.0002, *r* ^2^ = 0.84).

The number of both phrenic α motor axons and retrogradely labeled PhMNs was dependent on age (*F*_(1,16)_ = 52.2, *p* < 0.0001), but not dependent on confocal‐ or EM‐based analytical method (*F*_(1,16)_ = 2.0, *p* = 0.18; Two‐way ANOVA; Figure 1). Bonferroni post hoc tests revealed a 29% reduction in the number of phrenic α motor axons with age (young: 252 ± 37; old: 179 ± 41; *p* = 0.001; Figure 2a) and a 35% reduction in the number of retrogradely labeled PhMNs with age (young: 242 ± 27; old: 158 ± 9; *p* = 0.0009; Figure 2a). Note that we did not detect any difference between the number of phrenic α motor axons and retrogradely labeled PhMNs rats within the same age group (*p* > 0.99). Linear regression showed that within each rat, the number of phrenic α motor axons is almost at parity with the number of PhMNs (slope = 0.90, *p* = 0.0002, *r* ^2^ = 0.84), regardless of age (Figure 2b). Thus, assessment of changes in the number of phrenic α motor axons serves as a reliable alternative to assessing the number of PhMNs.

Importantly, the number of myelinated axons that were not classified as α motor axons (i.e., non‐α axons) were unchanged with aging (young: 104 ± 15; old: 119 ± 40; *p* = 0.35, Student's *t*‐test). Overall, the number of myelinated axons was unchanged with age (young: 356 ± 46; old: 304 ± 63; *p* = 0.12, Student's *t*‐test), consistent with past reports that did not distinguish phrenic α motor axons from non‐α myelinated axons (Smith & Rosenheimer, 1984).

### 3.3 Age‐related reduction in the number of larger PhMNs and phrenic α motor axons

**FIGURE 3:**
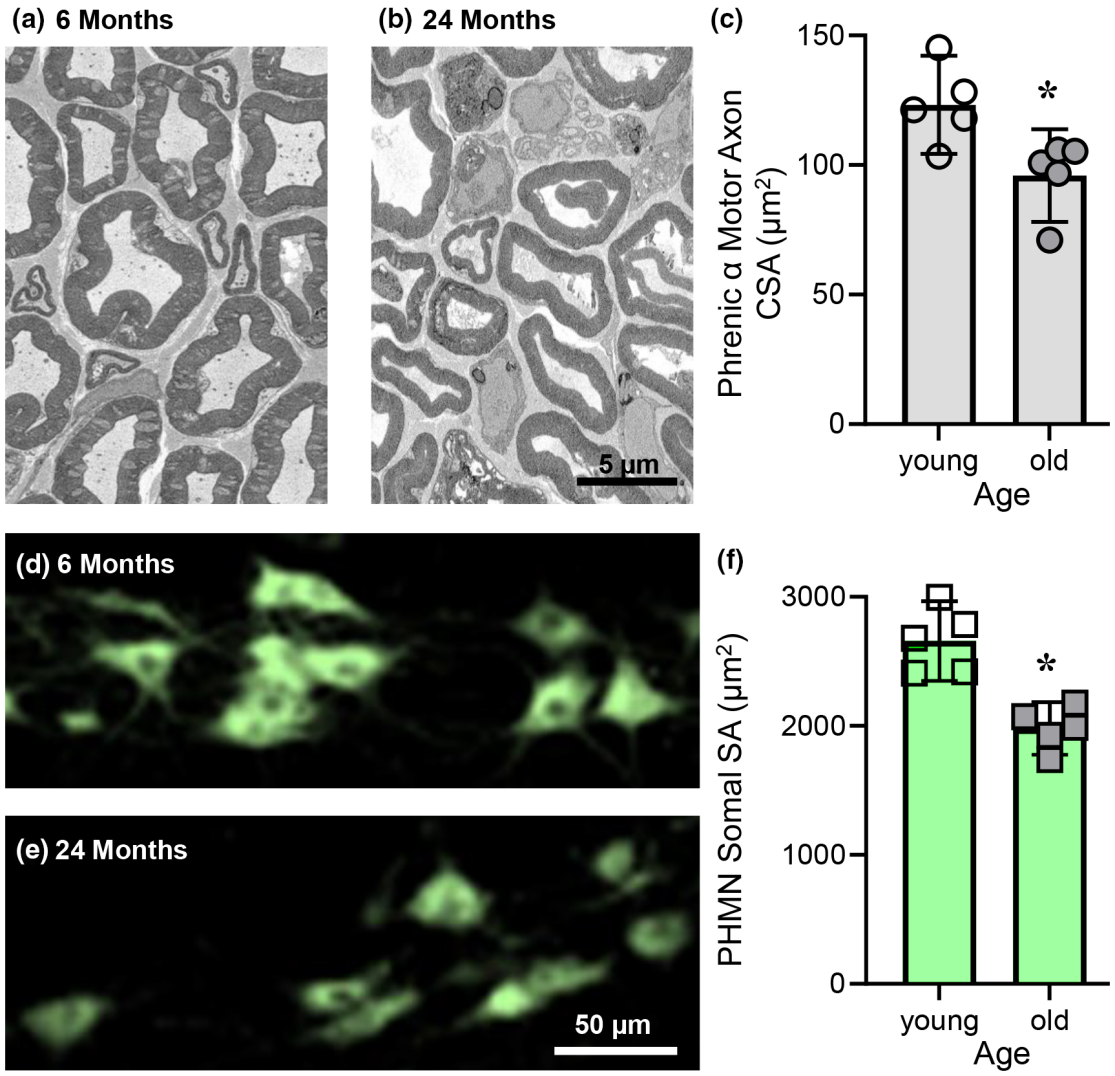
Reduced phrenic motor neuron (PhMN) somal surface area and phrenic α motor axon cross‐sectional area in old rats. (a, b) An example high‐magnification EM photomicrographs of phrenic α motor axons of young (6 months) and old (24 months) F344 rats, respectively. (c) Scatterplot shows reduced of phrenic α motor axon cross‐sectional area in old (dark gray circles) compared to young (open circles) F344 rats. (d, e) Example fluorescent photomicrographs of PhMNs of young (6 months) and old (24 months) F344 rats, respectively. (f) Scatterplot shows reduced PhMN surface area (SA) in old (dark gray squares) compared to young (open squares) F344 rats. Student's unpaired *t*‐tests, *p* < 0.05, *n* indicated by symbol. Each mean comprises high‐magnification PhMN SA estimates from a minimum of 30 (every 5th) PhMNs/rat/age and high‐magnification phrenic α motor axon CSA estimates from each counted axon.

The mean CSA of phrenic α motor axons was 22% smaller in older rats compared to younger animals (young: 123 ± 19 μm^2^; old: 96 ± 18 μm^2^; *p* = 0.019; Student's unpaired *t*‐test; Figure 3a–c). Based on 3‐D reconstructions of retrogradely labeled PhMNs, the mean somal surface area of PhMNs was 26% smaller in old rats compared to young animals (young: 2658 ± 310 μm^2^: 1980 ± 204 μm^2^; *p* = 0.001; Student's unpaired *t*‐test; Figure 3d–f).

**FIGURE 4:**
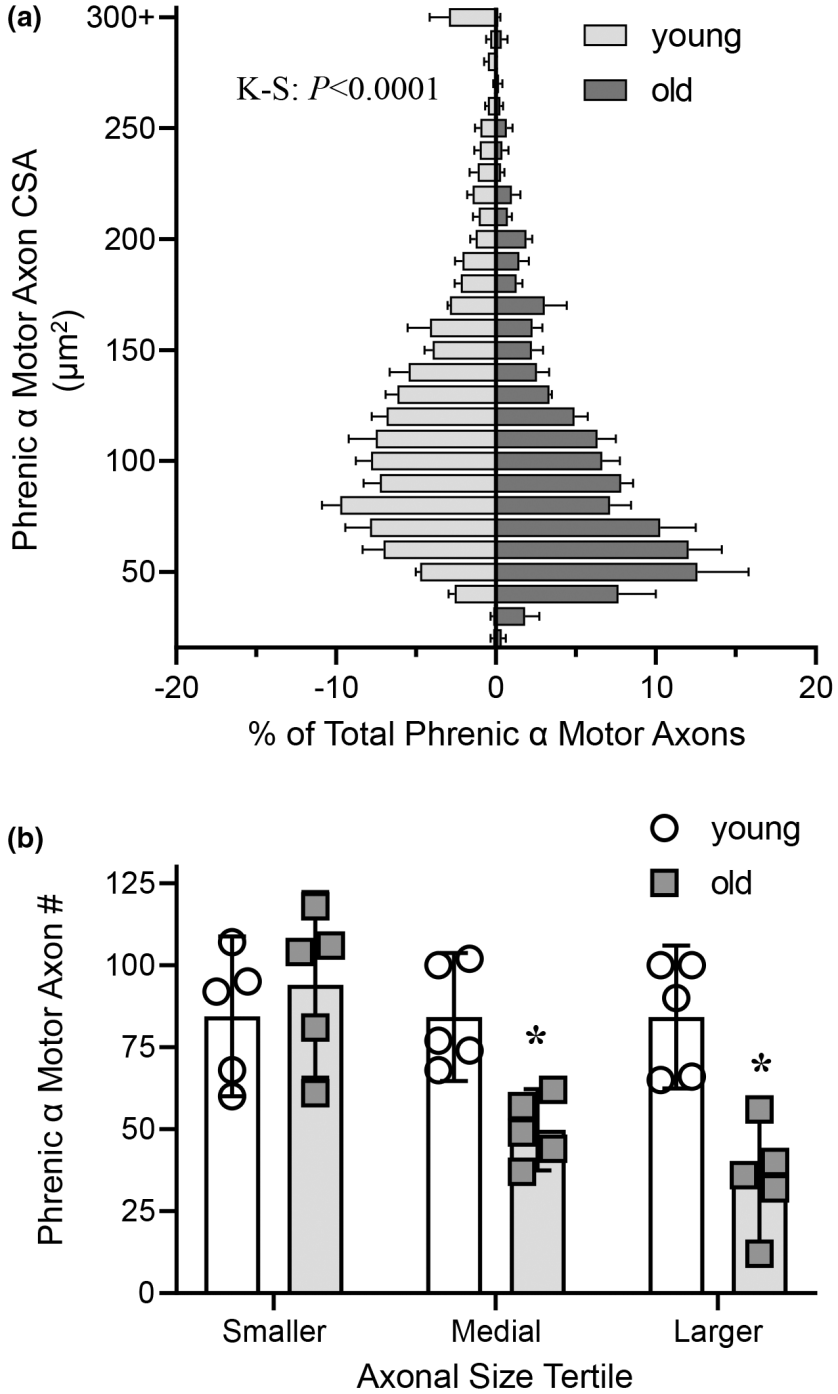
Larger phrenic α motor axons are disproportionately lost in old rats. (a) Reduced % of total phrenic α motor axons that are of larger size in old (dark gray bars) compared to young (light gray bars) F344 rats. Kolmogorov–Smirnov test, *p* < 0.0001. (b) Scatterplot shows reduced number of phrenic α motor axons in the medial and larger axon size tertile in old (24 months, squares), compared to young (6 months, circles) F344 rats. Two‐way ANOVA with Bonferroni post hoc test, *p* < 0.05, *n* indicated by symbol.

The frequency distribution of the CSAs of phrenic α motor axons differed between young and old rats (*p* < 0.0001, Kolmogorov–Smirnov; Figure 4a). Notably, there was a higher proportion of smaller α motor axons (i.e., CSAs <75 μm^2^) in 24‐month‐old rats compared to 6‐month‐old rats with a corresponding lower proportion of larger α motor axons (i.e., CSAs >225 μm^2^) in older rats compared to younger animals (Figure 4a).

When the number of phrenic α motor axons was stratified by CSA tertiles determined in 6‐month‐old rats, there were significant changes in the distribution of α motor axons in each size tertile that depended on age (*F*_(1,8)_ = 14.5, *p* = 0.005) and axon CSA tertile (*F*_(2,16)_ = 8.0, *p* = 0.004; Two‐way ANOVA; Figure 4b). Bonferroni post hoc tests revealed an ~40% reduction in the number of phrenic α motor axons in the middle tertile (young: 84 ± 20; old: 50 ± 12; *p* = 0.014) and an ~58% reduction in the number of phrenic α motor axons in the upper tertile (young: 84 ± 22; old: 35 ± 19; *p* = 0.0005) in older compared to younger rats (Figure 4b). The number of phrenic α motor axons in the lower tertile population did not differ with age (young: 84 ± 24; old: 94 ± 28; *p* > 0.99; Figure 4b).

### 3.4 Age‐related reduction in myelination of phrenic α motor axons

**FIGURE 5:**
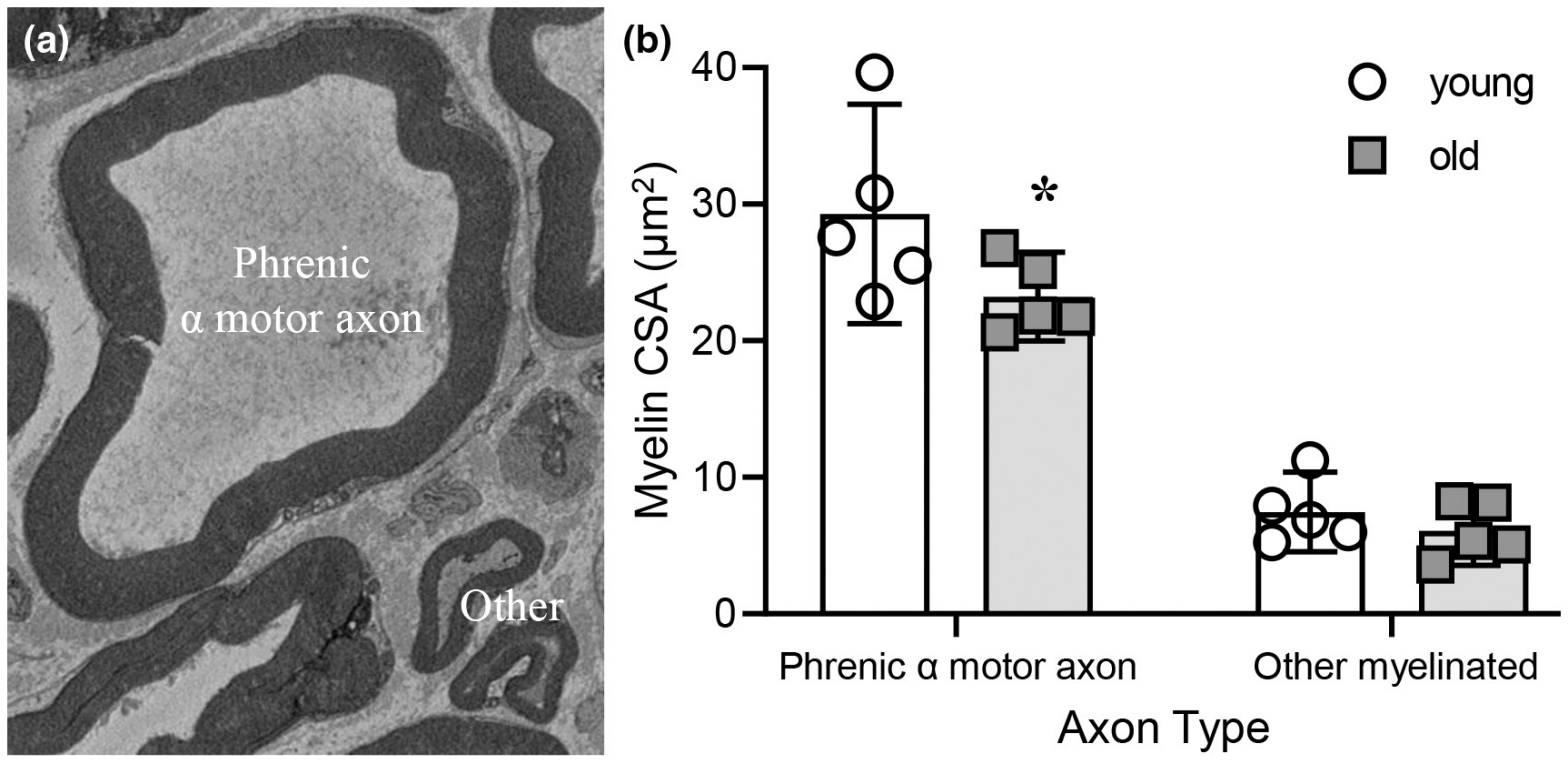
Reduced myelination in phrenic α motor axons in old rats. (a) An example high‐magnification EM photomicrograph of a phrenic α motor axon adjacent to other non‐α motor myelinated axons. (b) Scatterplot shows reduced mean myelination (μm^2^) of phrenic α motor axons in old (24 months, squares), compared to young (6 months, circles) F344 rats. Two‐way ANOVA with Bonferroni post hoc test, *p* < 0.05, *n* indicated by symbol. Each mean comprises high‐magnification myelin cross‐sectional area estimates from a minimum of 26 axons/rat/age.

The extent of myelination of phrenic α motor axons and non‐α myelinated axons was assessed by determining the proportion of the axon CSA that comprised the myelin sheath (Figure 5). The extent of myelination was dependent on age (*F*_(1,8)_ = 6.6, *p* = 0.034) and axon type (*F*_(1,8)_ = 100.8, *p* < 0.0001; Two‐way ANOVA; Figure 5b). Bonferroni post hoc tests revealed a reduction of ~20% in the myelination of old phrenic α motor axons (23.2 ± 3.0 μm^2^) compared to young (29.1 ± 7.4 μm^2^; *p* = 0.04), with myelination unchanged in non‐α myelinated axons (young: 7.5 ± 2.9 μm^2^; old: 6.1 ± 2.7 μm^2^; *p* > 0.99; Figure 5b). The overall extent of myelination was greater in phrenic α motor axons compared to non‐α myelinated axons, regardless of age (*p* < 0.01 for all combinations).

## 4 DISCUSSION

In the present study, we found a loss of larger PhMNs with aging in F344 rats corresponded to a similar loss of larger phrenic α motor axons. In addition, there was an indication that phrenic α motor axons exhibit decreased myelination with aging. These results are consistent with an age‐related loss of more fatigable FF motor units in the DIAm with aging while fatigue‐resistant type S and FR motor units, which are vital for breathing are preserved.

The results of the present study are important in the context of recruitment of DIAm motor units during different motor behaviors. The Size Principle provides the conceptual framework for the mechanism underlying motor unit recruitment. Although the Size Principle is generally considered with respect to the size of motor neurons (Fogarty, Mantilla, & Sieck, 2018; Fogarty & Sieck, 2019a; Gordon et al., 2004; Heckman & Enoka, 2012; Manuel et al., 2019), the original supporting evidence was based on differences in the conduction velocity of α motor axons, which is related to axon diameter (or CSA) (Clamann & Henneman, 1976; Dick et al., 1987; Henneman, 1957; Smith & Rosenheimer, 1984). Importantly, the original assumption that motor axon size corresponds to motor neuron size (Henneman, 1957), holds true empirically (Clamann & Henneman, 1976; Cullheim & Ulfhake, 1979; Dick et al., 1987) and is supported by the results of the present study. Size‐dependent features of motor neurons also encompass other properties such as the number and arborization of dendrites (Cullheim et al., 1987; Fogarty, Mu, et al., 2020; Fogarty, Mu, et al., 2021; Leroy et al., 2014; Ma & Vacca‐Galloway, 1991).

The size‐dependent recruitment of motor neurons reflects differences in membrane capacitance and input resistance, which are determined by motor neuron surface area (Henneman et al., 1965a, 1965b). In the present study, we show that the heterogeneity of PhMN surface areas is reflected in the heterogeneity of the diameter and CSA of phrenic α motor axons, with the range of all myelinated axons in the present study resembling that of prior studies (Gottschall, 1981; Langford & Schmidt, 1983; Mitteregger, 1979). Specifically in rats, the total number of myelinated phrenic α axons number found in the present study was within ~6%–19% of previous estimates (Smith & Rosenheimer, 1984). We identified phrenic α motor axons using two criteria: (i) axons must have a myelin sheath; and (ii) axons must have a diameter of >4 μm. Importantly, although it has been reported that axon diameters of PhMNs within the gray matter range from ~2.5 to 9 μm, other nerves that have been studied show enlargement of axons in the white matter and periphery, consistent with our size criteria (Fabricius et al., 1994). These criteria have been used in a variety of motor nerves, including the phrenic nerve (Cullheim, 1978; Fabricius et al., 1994; Landau et al., 1962), and are used extensively in the literature (Eccles & Sherrington, 1930; Gottschall, 1981; Henneman, 1957; Langford & Schmidt, 1983; Mitteregger, 1979; Stankovic et al., 2005). Although such criteria may be imperfect in identifying all α motor axons, these criteria are supported by the fact that the total number of phrenic α motor axons (mean: 252) was in excellent agreement (within 4%) of the number of retrogradely labeled PhMNs (mean: 242) in 6‐month‐old F344 rats.

Previously, we found that with aging, there is a loss of larger PhMNs, likely comprising FF motor units (Fogarty, Omar, et al., 2018). In that study, there were ~22% fewer PhMNs in 24‐month‐old F344 rats compared to 6‐month olds, with ~64% fewer PhMNs in the upper size tertile (determined in 6‐month‐old rats) of PhMNs (Fogarty, Omar, et al., 2018). In contrast in the present study, we found that there were ~34% fewer PhMNs in older rats. It should be noted that in our earlier study, PhMNs were labeled by dipping the cut phrenic in a solution containing rhodamine dextran (Brandenburg et al., 2020; Fogarty, Omar, et al., 2018; Mantilla et al., 2009). By contrast, in the present study, PhMNs were retrogradely labeled by intrapleural injections of CTB, which binds to gangliosides at the presynaptic terminal (Wolf et al., 1998). The CTB‐ganglioside complex is then internalized via endocytosis and transported by axoplasmic transport to the soma (Davis et al., 2022; Gonzalez Porras et al., 2016, 2019). Thus, CTB labeling depends on an intact presynaptic terminal, which may be affected by age‐related presynaptic terminal withdrawal (Fogarty, Gonzalez Porras, et al., 2019; Prakash & Sieck, 1998) and poor reinnervation of vacant muscle fibers (Aare et al., 2016; Einsiedel & Luff, 1992; Hepple & Rice, 2016). In this study we likely overestimated the age‐associated loss of PhMNs compared to nerve‐dip (Brandenburg et al., 2020; Fogarty, Omar, et al., 2018), where PhMNs are labeled via nerve dissection and are not affected by neuromuscular junction (NMJ) denervation (Brandenburg et al., 2020; Fogarty, Omar, et al., 2018; Mantilla et al., 2009). This interpretation is supported by the aged‐related change in the distribution of PhMN somal surface areas in the present study, which showed a greater proportionate loss of larger PhMNs than was found in our study using the phrenic nerve‐dip technique. This difference suggests that there is greater presynaptic terminal withdrawal and thereby decreased CTB uptake at NMJs innervating type IIx/IIb fibers (FF motor units) (Davis et al., 2022; Fogarty, Gonzalez Porras, et al., 2019).

In addition to our PhMN findings, age‐associated motor neuron loss has also been reported in the cervical (including the PhMN population) and lumbar motor columns in humans (Cruz‐Sánchez et al., 1998; Kawamura, O'Brien, et al., 1977; Tomlinson & Irving, 1977; Zhang et al., 1996) and rats (Hashizume & Kanda, 1990; Jacob, 1998; Rowan et al., 2012), and more particularly in the tibialis anterior innervating motor neurons of older rats (Ishihara et al., 1987). Curiously, frank motor neuron loss is not observed in aging mice, at least in the lumbar motor column of the C57/Bl6 strain (Blasco et al., 2020; Chai et al., 2011; Maxwell et al., 2018), although there is a report in BALB/C mice of a loss of large ventral horn neurons (presumably motor neurons) throughout the spinal cord (Wright & Spink, 1959). In mice, motor pool specific studies are lacking, perhaps due to difficulties of labeling discrete motor neuron pools in mice (Brandenburg et al., 2018, 2020). Regardless, with aging in both humans and rats, there is a loss of motor neurons, and that the associated denervation of muscle fibers may be a significant contributor to age‐associated sarcopenia.

In this study, we found an ~29% loss of phrenic α motor axons between 6‐ and 24‐month‐old F344 rats, generally reflecting the loss of PhMNs observed in the same animals. This age‐related loss of phrenic α motor axons generally matches the extent of PhMN loss found in our previous study (Fogarty, Omar, et al., 2018). It appears that the number of myelinated phrenic α motor axons is greater than the number of PhMNs within the same animal (Figure 2b). This is likely due to the fact that CTB does not label all PhMNs in older animals (Fogarty, Gonzalez Porras, et al., 2019). Alternatively, the greater loss of PhMNs compared to myelinated phrenic α motor axons may be related to “die‐forward,” whereby PhMN death occurs prior to denervation of DIAm fibers. In the present study, the total number of myelinated axons in the phrenic nerve did not change with age; an observation consistent with previous reports in F344 (Smith & Rosenheimer, 1984) and Sprague–Dawley (Inestrosa & Alvarez, 1988) rats. However, in these prior studies, there was no discrimination between myelinated phrenic α motor axons and non‐α axons, which we performed using well‐established morphometric axon diameter criteria (Eccles & Sherrington, 1930; Gottschall, 1981; Henneman, 1957; Langford & Schmidt, 1983; Mitteregger, 1979; Stankovic et al., 2005). Moreover, in other peripheral nerve studies which discriminated between α motor axons and non‐α axons, an age‐related loss of α motor axon loss has been reported in humans and rats (Ansved & Larsson, 1990; Kawamura, Okazaki, et al., 1977; Mittal & Logmani, 1987; Ochoa & Mair, 1969; O'Sullivan & Swallow, 1968; Tohgi et al., 1977). Regardless of methodological differences, an assessment of the overall number of myelinated axons was complicated by the greater variability in the number of myelinated non‐α motor axons (coefficient of variation: young = 26%; old = 25%), compared to the variability in the number of phrenic α‐motor axons (coefficient of variation: young = 11%; old = 19%). One interpretation is that there is an increase in myelinated non‐α motor axons (including γ motor neurons and sensory neurons) with age, although γ motor neurons are scarce in the DIAm and any differences in functional peripheral sensation seem equivocal with age (Caetano et al., 2022; Kiernan et al., 2001). Alternatively, it is possible that there is an age‐related atrophy/degeneration of phrenic α‐motor axons such that there size falls below the CSA threshold. This would be consistent with results in lumbar ventral roots of old mice (Chung et al., 2017).

The observation of an ~22% reduction in the mean CSA of phrenic α motor axons in older F344 rats is consistent with the loss of larger PhMNs found in the present and previous study (Fogarty, Omar, et al., 2018). By contrast, a prior study in Sprague–Dawley rats did not find an age‐related reduction in either mean CSA or diameter of unclassified myelinated axons between 3 and 4 month‐old animals compared to 24‐month‐old rats (Inestrosa & Alvarez, 1988). Similarly, when comparing 10‐month‐old F344 rats to 24–28‐month‐old animals (Smith & Rosenheimer, 1984) no difference in mean CSA or diameter of myelinated axons was found. Importantly, in the study using Sprague–Dawley rat, studies in other peripheral nerves has shown that axons continue to grow and only reach maximum diameters between 6 and 9 months of age (Sharma et al., 1980). Unfortunately, age‐related changes in myelinated axon CSAs in many peripheral nerves are inconsistent, with occasional reports of increased axon size (Blasco et al., 2020; Inestrosa & Alvarez, 1988) contrasting the majority reporting smaller myelinated axon size (Ansved & Larsson, 1990; Ceballos et al., 1999; Jacobs & Love, 1985; Jeronimo et al., 2008; Knox et al., 1989; Mortelliti et al., 1990; O'Sullivan & Swallow, 1968; Sakita et al., 2016; Sharma et al., 1980; Soltanpour et al., 2012; Tohgi et al., 1977; Ugrenović et al., 2016). Despite these vagaries, there are consistent findings of atrophy of myelinated axons in humans (Jacobs & Love, 1985; Mortelliti et al., 1990; O'Sullivan & Swallow, 1968; Tohgi et al., 1977; Ugrenović et al., 2016) and F344 rats (Knox et al., 1989; Sharma et al., 1980), highlighting the utility of this strain for human gerontology. It is also likely that the matching between size‐dependent motor neuron loss and axonal loss is strain and pool dependent, in addition to differences in methodology (Smith & Rosenheimer, 1984), even within a single nerve from an individual rat (Fraher, 1992).

Overall, the loss of larger PhMNs and α motor axons in aged F344 rats is entirely consistent with associated behavioral, functional and anatomical deficits. Specifically these larger PhMNs and α motor axons comprise FF motor units that produce the greatest forces (Geiger et al., 2000) and transdiaphragmatic pressures (Pdi) (Fournier & Sieck, 1988) when activated, while smaller type S and FR motor units produce lower Pdi in an indefatigable manner (Fogarty & Sieck, 2019a). In old F344 rats, fatigue‐resistant breathing behaviors are conserved (Fogarty, Mantilla, & Sieck, 2019; Fogarty, Marin Mathieu, et al., 2020; Khurram et al., 2018), with higher force maximum effort behaviors impaired (Khurram et al., 2018). Similar patterns of resilience and vulnerability in DIAm fibers of F344 rats are found, with no atrophy of type I or IIa fibers, and substantial reductions in the CSA of type IIx/IIb DIAm fibers with age, along with concomitant reduction in maximum isometric force (Fogarty, Mantilla, & Sieck, 2019; Fogarty, Marin Mathieu, et al., 2020; Gosselin et al., 1994; Khurram et al., 2018). Impairments in DIAm NMJs with aging in F344 rats are also selective for FF motor units, with neuromuscular transmission failure and NMJ pre‐ and postsynaptic overlap reduced (Fogarty, Gonzalez Porras, et al., 2019; Prakash et al., 1993; Smith, 1979; Smith & Rosenheimer, 1982).

The myelin thickness of phrenic α motor axons was smaller in old age. These findings are consistent with anatomical studies of myelinated axons in other peripheral nerves in humans, mice and rats (Ceballos et al., 1999; Jacobs & Love, 1985; Knox et al., 1989; Sakita et al., 2016; Soltanpour et al., 2012; Ugrenović et al., 2016) and with functional conduction impairments (Campbell et al., 1973; Kanda et al., 1986; Norris et al., 1953; Sato et al., 1985) including of phrenic nerves (Imai et al., 2005; MacLean & Mattioni, 1981; McKenzie & Gandevia, 1985) in aging studies. However, in aged F344 rats, an absence of nerve conduction velocity changes coincided with unchanged myelinated axon thickness (Smith & Rosenheimer, 1984) (using a differing methodology), although distinguishing phrenic α motor axons from non‐α myelinated axons was not attempted. Taken together, the results from the present and past studies in F344 rays suggest that demyelination is not as substantial a problem as either frank PhMN loss (Fogarty, Omar, et al., 2018) or the peripheral effects of DIAm NMJ denervation, observed by multiple groups in phrenic nerve‐DIAm preparations (Fogarty, Gonzalez Porras, et al., 2019; Lee et al., 2017; Prakash & Sieck, 1998; Smith, 1979; Smith & Rosenheimer, 1982).

In conclusion, we report a robust agreement between the number of labeled PhMNs and the number of phrenic α motor axons in young and old F344 rats. With aging, we observe a disproportionate loss of larger PhMNs, reflected in the smaller number and CSA of phrenic α motor axons in old F344 rats. We also show that a demyelination of phrenic α motor axons in old F344 rats may contribute to the plethora of functional DIAm deficits (Fogarty, Gonzalez Porras, et al., 2019; Fogarty, Mantilla, & Sieck, 2019; Gosselin et al., 1994; Khurram et al., 2018; Smith, 1979) that plague aged F344 rats. Motor axon assessment may also prove a useful approach in conditions where reliable labeling of MN pools is technically challenging (e.g., in mice) or precluded (e.g., embryonic/early postnatal).

## AUTHOR CONTRIBUTIONS

MJF and GCS contributed to study design. MJF performed the experiments and analyzed the data. MJF wrote the first draft of the manuscript. MJF and GCS, revised the manuscript and figures. All authors approved the final version of the manuscript.

## FUNDING INFORMATION

Funding for this research was provided by NIH (R01‐AG44615) to GCS.

## CONFLICT OF INTEREST

The authors declare that there is no real or perceived conflict of interest.

## ETHICS STATEMENT

All protocols were approved by the Mayo Clinic IACUC (#57714) and complied with national and international guidelines.
